# Strategies for Using Postcolumn Infusion of Standards to Correct for Matrix Effect in LC-MS-Based Quantitative Metabolomics

**DOI:** 10.1021/jasms.4c00408

**Published:** 2024-11-15

**Authors:** Anne-Charlotte Dubbelman, Bo van Wieringen, Lesley Roman Arias, Michael van Vliet, Roel Vermeulen, Amy C. Harms, Thomas Hankemeier

**Affiliations:** % Metabolomics and Analytics Centre, Leiden Academic Centre for Drug Research, Leiden University, Einsteinweg 55, 2333 CC Leiden, The Netherlands; & Institute for Risk Assessment Sciences, Utrecht University, 3508 TC Utrecht, The Netherlands

## Abstract

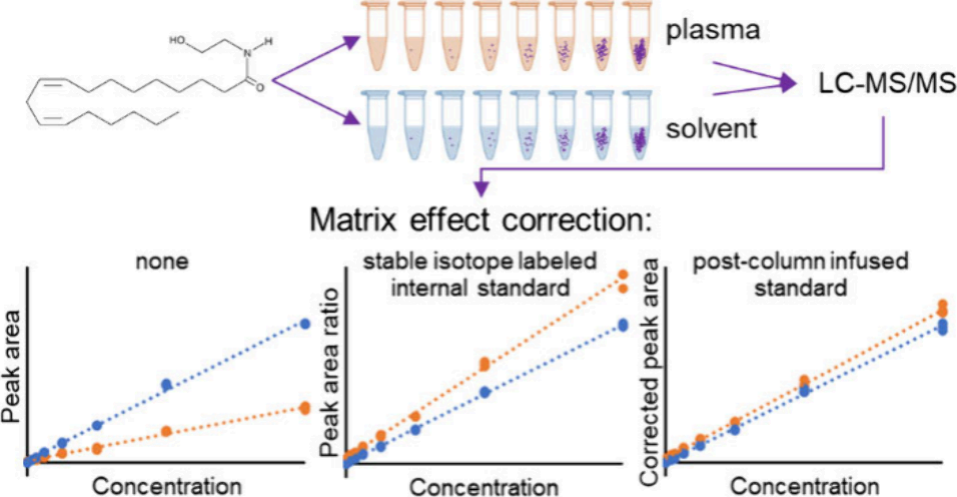

The matrix effect limits the accuracy of quantitation of the otherwise popular metabolomics technique liquid chromatography coupled to mass spectrometry (LC-MS). The gold standard to correct for this phenomenon, whereby compounds coeluting with the analyte of interest cause ionization enhancement or suppression, is to quantify an analyte based on the peak area ratio with an isotopologue added to the sample as an internal standard. However, these stable isotopes are expensive and sometimes unavailable. Here, we describe an alternative approach: matrix effect correction and quantifying analytes using a signal ratio with a postcolumn infused standard (PCIS). Using an LC-MS/MS method for eight endocannabinoids and related metabolites in plasma, we provide strategies to select, optimize, and evaluate PCIS candidates. Based on seven characteristics, the structural endocannabinoid analogue arachidonoyl-2′-fluoroethylamide was selected as a PCIS. Three methods to evaluate the PCIS correction vs no correction showed that PCIS correction improved values for the matrix effect, precision, and dilutional linearity of at least six of the analytes to within acceptable ranges. PCIS correction also resulted in parallelization of calibration curves in plasma and neat solution, for six of eight analytes even with higher accuracy than peak area ratio correction with their stable isotope labeled internal standard, i.e., the gold standard. This enables quantification based on neat solutions, which is a significant step toward absolute quantification. We conclude that PCIS has great, but so far underappreciated, potential in accurate LC-MS quantification.

## Introduction

The field of metabolomics, which studies small molecules in biological matrices, is rapidly growing and the number of published studies reporting on molecular biomarkers with e.g. diagnostic or prognostic value continues to increase.^1^ While individual metabolomics studies can provide valuable information, the potential of metabolomics could be further exploited if data of different studies could be combined and interpreted beyond their original goals. This is currently challenging due to the usually semiquantitative nature of metabolomics data, hampering its widespread translation to the clinic and integration with other -omics data.^2^

The most widely applied technique in metabolomics is liquid chromatography coupled to mass spectrometry (LC-MS).^3^ This technique has a superb sensitivity compared to e.g. NMR, but it is not as quantitative.^4^ Absolute quantification using LC-MS requires customized methods using calibration lines with reference standards. Usually, internal standards (ISTDs) are added to all samples, including calibration samples, to correct for (i) variability in analyte recovery during sample preparation and (ii) variability of the effect of other components in the sample matrix on the ionization of the analyte-of-interest (matrix effect). Quantification is then performed using the peak area ratio of the analyte and its ISTD. The latter can be a structural analogue of the analyte-of-interest, of which successful cases have been reported,^5^ but the ideal ISTD is a stable isotope-labeled (SIL) analogue because of its near identical chemical and physical characteristics.^6^ However, these SIL standards are usually expensive and their commercial availability is limited.^5^

The costs and poor availability of SIL standards, but also the hassle of adding individual ISTDs per analyte are some of the reasons that increasing the metabolite coverage of a method is often at the expense of the quantitative reliability.^7^ This, because in practice methods with high metabolic coverage often only use one or two ISTDs to correct for the recovery and matrix effect of a whole compound class.^8^ Although it may be justified that this suffices to correct for variation in recovery, limited ISTDs may not be adequate for the correction of matrix effect during ionization. Matrix effect can be caused by compounds coeluting with the analyte of interest by e.g. affecting the droplet formation, changing the droplet viscosity or surface tension, competing for charge, or inducing coprecipitation with nonvolatiles.^9^ These processes change with the composition of the eluent exiting the LC column, and an ISTD not coeluting with the analyte of interest can seriously compromise quantification.

A solution to this problem of noncoeluting ISTDs was already proposed by Choi et al. in 1999.^10^ They demonstrated that by continuously adding a SIL drug after the LC column and before entering the MS, as a postcolumn infused standard (PCIS), its signal could correct for ion suppression on both the drug and its earlier eluting metabolite. This was not possible when the same compound was added to the samples as a spiked ISTD, because the two analytes suffered from ion suppression to a different extent.^10^ After this first publication, the innovative PCIS technique for matrix effect correction has only sparsely been adopted,^11−16^ even though it has great potential to overcome practical issues in quantitation.

The limited use of PCIS might be explained by an underappreciation of the problem of matrix effect, a reluctance to abandon the widely recognized method for matrix effect correction (i.e., adding SIL-ISTDs to all samples), an underestimation of the great potential of PCIS or a lack of guidance on how to incorporate PCIS in existing or new LC-MS methods. With the current paper, we aim to remove these possible objections and demonstrate that PCIS is a solution to elevate metabolomics from semiquantification to absolute quantification. Using an LC-MS/MS method for measuring metabolites related to the endocannabinoid system in plasma, for which SIL standards were available and which suffer from ion suppression,^17^ we will provide strategies to incorporate PCIS into LC-MS/MS methods. We will discuss proposed characteristics of a good PCIS candidate and the optimization of its concentration. Various approaches to evaluate the PCIS performance will be demonstrated and processes for which a PCIS cannot correct will be discussed. Finally, we will show the potential of PCIS as a replacement of SIL-ISTDs and as a tool enabling absolute quantification based on a calibration curve of standards in neat solution instead of matrix.

## Experimental Section

### Chemicals and Biologicals

LC grade acetonitrile (ACN), ULC grade isopropyl alcohol (IPA), ULC grade methanol absolute (MeOH) and ULC grade ethanol absolute (EtOH) were purchased from Biosolve (Valkenswaard, The Netherlands). Acetic acid, ethylenediaminetetraacetic acid (EDTA), butylated hydroxytoluene (BHT), 1-Butanol (BuOH) and MTBE were obtained from Sigma-Aldrich (Zwijndrecht, The Netherlands). Citric acid (monohydrate) and disodium hydrogen phosphate were purchased from Merck (Darmstadt, Germany), as was the Ultrapure Milli-Q system which was used for water. Both the unlabeled standards (linoleoyl ethanolamide (LEA), docosahexaenoyl ethanolamide (DHEA), arachidonoyl ethanolamide (AEA), N-arachidonoyl dopamine (NADA), palmitoyl ethanolamide (PEA), 1-arachidonoyl glycerol (1-AG), 2-arachidonoyl glycerol (2-AG), oleoyl ethanolamide (OEA) and stearoyl ethanolamide (SEA)), the deuterated standards (linoleoyl ethanolamide-d4 (d4-LEA), docosahexaenoyl ethanolamide-d4 (d4-DHEA), arachidonoyl ethanolamide-d8 (d8-AEA), N-arachidonoyl dopamine-d8 (d8-NADA), palmitoyl ethanolamide-d4 (d4-PEA), 2-arachidonoyl glycerol-d8 (d8–2-AG), oleoyl ethanolamide-d4 (d4-OEA) and stearoyl ethanolamide-d3 (d3-SEA), and the PCIS standard arachidonoyl-2′-fluoroethylamide (2F-AEA) were obtained from Cayman Chemical (Ann Harbor, MI, USA, via SanBio, Uden, The Netherlands). The SI (Table S1) gives an overview of the structures and classification^18^ of the used compounds. K2EDTA plasma from 9 diverse healthy donors was purchased from BioIVT (Westbury, NY, USA). A K_2_EDTA plasma pool of 10 donors, here referred to as lab plasma pool, was obtained from Innovative Research, Inc. (Novi, MI, USA).

### Sample Preparation

Samples were prepared by liquid–liquid extraction according to a validated method^19−21^ with slight optimization. Briefly, 150 μL plasma aliquots were (on ice) mixed with 5 μL of antioxidant solution (0.4 mg/mL BHT in MeOH: 0.4 mg/mL EDTA in water, 1:1 v:v), 40 μL of 0.4 mg/mL BHT solution in MeOH, 150 μL of a 0.2 M citric acid and 0.4 M disodium hydrogen phosphate buffer in water, and 1 mL of BuOH:MTBE (1:1 v:v). After resting for 20 min on ice, they were mixed in a bullet blender (2 min, highest speed) and centrifuged (10 min, 4 °C, 15700 g). A volume of 850 μL of the upper organic phase was transferred to a 1.5 mL Eppendorf tube and dried overnight in a SpeedVac (ThermoFisher Savant) at 30 °C. The dried samples were reconstituted in 50 μL of ice-cold injection solution (90% MeOH, 10% Milli-Q, 100 nM CUDA) to prepare blank plasma or in 50 uL of a combination of the injection solution, (deuterated) standard mix and 0.4 mg/mL BHT in MeOH. Samples were vortex mixed and centrifuged (10 min, 4 °C, 15700 g) prior to being transferred to an autosampler vial with an insert.

### Preparation of Standards and Mixes

From the endocannabinoid reference standards or solutions, individual stock solutions were prepared of 1 mM in ACN (1-AG and 2-AG), 1 mM in EtOH (LEA, DHEA, AEA, PEA and SEA) or 0.1 mM in EtOH (NADA). A mixed calibration stock solution of unlabeled standards was prepared in ACN with concentrations of 27 μM of PEA, 13.5 μM of LEA, DHEA, AEA, 1-AG, 1-AG, OEA and SEA, and 1.35 μM of NADA. This solution was 18 times diluted to obtain the CAL9 solution, which was serially diluted 1:1 v:v with 0.4 mg/mL BHT in MeOH, to obtain CAL8, CAL7 etc. until CAL2.

Individual stock solutions of each deuterated endocannabinoid were made in ACN at 0.10 mM, only for 2AG-d8 at 0.26 mM. A mixed stock solution of deuterated standards was prepared by mixing the right volumes of each individual stock and diluting with ACN to concentrations of 6.25 μM for all deuterated analytes, except for d8-NADA which had a concentration of 0.625 μM. This stock mix was further diluted with 0.4 mg/mL BHT in MeOH to a mixed working solution with concentrations of 0.188 μM (and 10 times lower for d8-NADA).

### LC-MS/MS Analysis and Postcolumn Infusion

Chromatographic separation and MS/MS analysis was performed according to the low pH method described by Di Zazzo et al.,^21^ using the same ternary mobile phase system and column (Acquity BEH C18 column (50 mm × 2.1 mm, 1.7 μM) (Waters, Milford, MA, USA)), but transferred to a Shimadzu Nexara X2 UHPLC system (Shimadzu, Kyoto, Japan) coupled to a Sciex QTRAP 6500+ MS (Sciex, Framingham, MA, USA) with electrospray ionization (ESI). The injection volume was 10 μL and mobile phase A was used as a stacking solution to avoid breakthrough. While the method was developed and validated to analyze a variety of metabolic classes, including oxylipins, oxidative stress markers and bile acids, this study only investigated endocannabinoids. The LC and MS method details can be found in the SI (Table S2). Because of uncontrolled isomerization between 1-AG and 2-AG, isomer peaks of 1-AG and 2-AG were integrated as a double peak (and referred to as 1/2-AG). Also d8–2AG was integrated as a double peak.

Postcolumn infusion was done using either the QTRAP built-in syringe pump with a 1.0 mL Hamilton syringe (Sigma-Aldrich, Saint Louis, USA) (for matrix effect exploration and PCIS concentration optimization) or an Agilent 1260 Infinity pump (Agilent Technologies, Santa Clara, USA) with a degasser (for all other experiments). Unless otherwise indicated, the flow rate was set at 50 μL/min. The flows were combined after the divert valve and before entering the MS using a T-piece (Upchurch Scientific, Idex Health and Science LLC, Oak Harbor, WA, USA). A schematic drawing of the instrument setup is included in the SI (Figure S1).

### Exploration of Matrix Effect

To explore the extent of matrix effect in our example method, the deuterated endocannabinoid working solution was diluted 1:1 v/v with 50% ACN in water and infused postcolumn by syringe, while injecting a plasma sample applying the LC-MS conditions as described above. The MRM transitions of the infused deuterated standards were monitored throughout the whole chromatogram.

### PCIS Concentration Optimization

To optimize the PCIS concentration, solutions of 25, 50, 100, 200, and 300 ng/mL 2F-AEA were postcolumn infused (at 0.05 mL/min) for 2 min using isocratic mobile phase conditions reflecting the gradient composition at 1 min (73% A, 26% B, 1% C, 0.7 mL/min). The signal of the 2F-AEA MRM transition (*m*/*z* 350.3 to 269.2) between 0.2 and 1.8 min was exported to Excel, smoothed (3-point moving average) and used to calculate the mean intensity and signal stability (relative standard deviation, RSD).

The same solutions were postcolumn infused while injecting a plasma sample (fortified with the CAL4 mix of nonlabeled endocannabinoids) on the column, running the described gradient separation. PCIS induced matrix effects were calculated as the mean ratio of peak areas in plasma while infusing each PCIS solution versus infusing plain ACN, whereby the latter was performed in triplicate.

A lower limit of quantification (LLOQ) for each PCIS signal was set at either 1% of the mean PCIS signal intensity during infusion (at isocratic conditions) or 10 times the mean noise measured over the first 0.5 min of the plasma injections (when the PCIS solution was still diverted to waste), whichever was lowest. The number of data points below LLOQ of the smoothed signal between 10 and 14 min were counted for each PCIS concentration.

### PCIS Data Preprocessing

Raw vendor data files (.wiff) were converted to mzML files using MSConvert (v. 3.0, Proteo Wizard^22^). A script was developed for peak integration for LC-MS/MS based experiments with PCIS correction. As input, the script requires the mzML files, a list of targets and a list of PCISs with their parameters. Using the target list, the software detects and extracts the MRM signals, followed by resampling and smoothing. It calculates PCIS corrected signals as the scan-by-scan ratio of the analyte signal and the PCIS signal, and peak areas as the sum of data points within a specified height of the peak (here set at 99% below maximum). It outputs a data report, including peak areas and PCIS corrected peak areas, as well as pdf-files with chromatograms of before and after correction to enable checking the peak integration and, if necessary, adapting input parameters. The script and a set of demo files have been published in the Github repository github.com/leidenuniv-lacdr-abs/endoc_pciis. Further data processing was done in Excel (Microsoft).

### Evaluating the Ability of PCIS to Correct for Differences in Matrix Effect

Three methods were applied to test the performance of 2F-AEA as a PCIS. For Method 1, 9 diverse plasma samples (numbered 1 to 9) and an aliquot of the lab plasma pool were prepared. They were reconstituted in 50 μL, consisting of 30 μL of injection solution, plus 5, 10, or 20 μL of mixed working solution of deuterated standards and 15, 10, or 0 μL of 0.4 mg/mL BHT in MeOH to prepare concentration levels at LOW (18.8 nM of d4-LEA, d4-DHEA, d8-AEA, d8–2-AG, d4-OEA, d3-SEA and d4-PEA and 1.88 nM of d8-NADA in plasma 1, 2 and 3), MID (37.6 nM of d4-LEA, d4-DHEA, d8-AEA, d8–2-AG, d4-OEA, d3-SEA and d4-PEA and 3.76 nM of d8-NADA in plasma 4, 5, 6 and the lab plasma pool) and HIGH (75.2 nM of d4-LEA, d4-DHEA, d8-AEA, d8–2-AG, d4-OEA, d3-SEA and d4-PEA and 7.52 nM of d8-NADA in plasma 7, 8, 9) concentration, respectively. For each level, a neat (matrix-free) solution was prepared identically. Samples were analyzed in triplicate using the described LC-MS/MS method with 2F-AEA (150 ng/mL) as a PCIS.

For Method 2 and 3, 8 150-μL-aliquots of the lab plasma pool were spiked with 20 uL of the CAL4 solution of endocannabinoids and prepared until dryness. They were reconstituted in 24 μL of injection solution (concentration factor (CF) 6.25), pooled and distributed over vials to create a dilution series containing 0, 2, 4, 8, 16, 24, 32, or 40 μL of CF 6.25 plasma, 10 μL of mixed working solution of deuterated standards and 40, 38, 32, 24, 16, 8, or 0 μL of injection solution, respectively. This resulted in plasma samples with a CF ranging from 5 to 0.25, which were analyzed in triplicate using LC-MS/MS with 2F-AEA as a PCIS. In this study, the same set of samples was used for Method 2 and 3. However, as the deuterated standards are not being used in the calculations of Method 3, samples for this method could also have been prepared without addition of the SIL standards.

Evaluation of the ability of the matrix effect correction by the PCIS was in Method 1 done for each SIL-ISTD by dividing its peak area in each sample by its peak area in the neat solution at the MID concentration, with and without PCIS correction. In method 2 it was done for each SIL-ISTD by dividing its peak area in a plasma sample with a certain CF by its peak area in the neat solution, both with and without PCIS correction. In method 3, the evaluation was done for each nonlabeled compound-of-interest by (i) linearly fitting the peak areas over the plasma CF and determining the coefficient of determination and (ii) by calculating the coefficient of variation of the ratios of the peak area and the plasma CF, again both with and without PCIS correction.

### From Relative to Absolute Quantification

To test whether the PCIS 2F-AEA enables endocannabinoid quantification using a calibration curve in neat solution, 12 aliquots of the lab plasma pool were prepared until dryness, reconstituted in 24 μL of injection solution and pooled. A plasma calibration curve was created mixing 20 μL of plasma pool with 20 μL of a CAL-solution (CAL2 up to CAL8) and 10 μL of mixed working solution of deuterated standards. Also, a blank (nonspiked plasma) was included, wherein 0.4 mg/mL BHT in MeOH replaced the CAL-solution. Neat calibration standards were prepared similarly, only the plasma concentrate was replaced by injection solution. Samples were analyzed in duplicate using LC-MS/MS with 2F-AEA as a PCIS.

## Results and Discussion

This study aims to demonstrate how postcolumn infusion of standards (PCIS) can be used to move toward absolute quantification in metabolomics LC-MS methods. Although the example that we use to illustrate this is based on LC-MS/MS, the strategies can also be applied to LC-high resolution mass spectrometry (LC-HRMS).

To incorporate PCIS into LC-MS methods we use 4 steps: exploration of the extent of matrix effect during ionization, selection of candidate analytes for PCIS, optimizing the concentration of the PCIS solution and evaluating the PCIS correction. Each of these steps will be discussed in the following sections.

### Exploration of Matrix Effect

In LC-MS analyses, postcolumn infusion of analytes-of-interest while injecting a matrix sample is a common procedure to get insight in how and when the analytes are affected by matrix effect.^23^ To assess the potential of PCIS correction for a given LC-MS method, it is useful to start by infusing a mix of analytes-of-interest and exploring the infusion profiles. Even though PCIS correction can in general be helpful to correct for any unexpected sources of matrix effect, it will be particularly useful for methods with analytes eluting in known high-suppression zones. Next to providing information on the extent of matrix effect, we propose using the infusion experiment data to estimate the number of different PCISs necessary to correct for the complete set of analytes. It is known that an analyte might be more or less prone to matrix effect depending on its ionization energy and proton affinity.^24^ Also other (physicochemical) properties have been reported in models predicting ionization efficiencies.^25^

**Figure 1:**
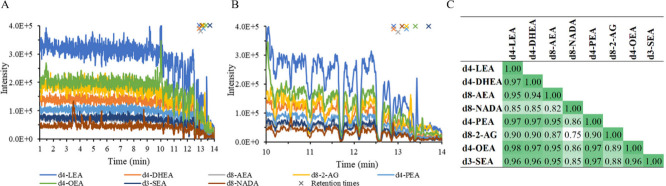
Exploring the matrix effect and comparing the PCIS profiles of deuterated endocannabinoids. (A) Overlaid MRM traces of infused stable isotope labeled endocannabinoids when injecting plasma, with the retention time of each endocannabinoid marked with an “x” in the same color. A severe ion suppression zone starts around 10 min. (B) Zoomed in on the region with severe ion suppression. (C) Overview of the Pearson correlation coefficients between the signals of the deuterated endocannabinoids in the 10.5–14 min region.

It could be expected that analytes with similar ionization efficiencies can be corrected with a single PCIS. This is exemplified with the method for endocannabinoids, for which the result of the matrix effect exploration is visualized in Figure 1A. Notably, using stable isotope labeled standards for this exploration is beneficial as it avoids endogenous analyte peaks in the matrix effect profiles. Figure 1A shows that the PCI profiles are all relatively stable between 1 and 10 min. However, as more clearly visible in Figure 1B, the signals drop frequently during the last 4 min, and this high-suppression zone coincides with the elution region of our analytes. The variability in the matrix effect over the last minutes of the run shows that quantification would be compromised if only one or two ISTDs would be spiked to a sample to correct the whole set of analytes for matrix effect and that this method could benefit from PCIS correction.

The similarity of the response to matrix effect was assessed by calculating the Pearson correlation coefficients of the signals of the infused standards along the high-suppression zone of the LC-MS/MS run (10.5–14 min). The table in Figure 1C shows that d8-NADA and d8–2-AG had correlation coefficients below 0.91 with all other analytes, while for the remaining analytes the correlation was mostly >0.95. In addition, the signals of d8-NADA and d8–2-AG did not correlate well with each other (0.75). From a structural perspective, a difference in ionization between most endocannabinoids and d8–2-AG, which lacks the amide group, might have been expected. For d8-NADA it was suspected that the low correlation was partially influenced by other compounds with the same transition and by its overall low intensity.

Based on these results of this simple infusion experiment and assuming a 0.95 correlation between signals is sufficient to share a PCIS, it can be estimated that 3 PCIS compounds would be needed to correct for the matrix effect on these 8 targets, one for 2-AG, one for NADA and one for the others.

### Selection of a PCIS Candidate

To successfully correct for matrix effects in LC-MS/MS analysis and to be applied in routine analysis, we here propose a set of characteristics that a PCIS candidate should possess. A high-potential PCIS candidate:

1. Is similar in its response to matrix effect as the analyte-of-interest. Obviously, in this respect a stable isotope (having the same physicochemical properties) would be ideal. Still, their limited availability and cost leads to the next best option, which is a structural analogue. The traditional disadvantage of structural analogues (not necessarily coeluting with the analyte and therefore experiencing different ionization conditions) is overcome by the continuous PCIS signal over time.
2. Is commercially available and affordable. It should be considered that the consumption of ISTD per sample will be higher if it is infused as compared to if it is added to the sample.
3. Ideally, has a commercially available and affordable SIL, which can be used as a spiked ISTD to correct for sample preparation recovery or injection volume. Since a spiked ISTD can also suffer from differences in matrix effect between samples, it should also be corrected by a PCIS. To prevent multiplication of errors, it is even more important for a spiked ISTD that the similarity in response to matrix effect between ISTD and PCIS is very close to perfect (as a stable isotope would be).
4. Is stable in solution for at least the time of an analysis batch at lab temperature (or, if necessary, with appropriate cooling). Namely, degradation of a PCIS during a batch would result in an increasing overcorrection.
5. Is measurable with a specific signal. When measuring in multiple reaction monitoring (MRM) mode, it should have a selective MRM transition, giving limited to no signal in matrix without infusing the PCIS candidate and should not interfere with the MRM transitions of the analytes. The presence of a nonendogenous atom, like fluorine, can help in achieving that.
6. Is easily fragmentable to the selective fragment. The most selective MRM transition may not be the most sensitive MRM transition and might require a higher concentration to give a stable PCIS signal. However, as discussed in the next section, a too high PCIS concentration can cause (significant) matrix effect by itself, resulting in a lower sensitivity for the analytes-of-interest
7. Preferably has [M + H]^+^ or [M-H]^−^ as the main ion with limited adduct formation or in-source fragmentation. This is because using an adduct may cause a higher variability in signal^26^ and if adduct formation is significant, the signal of the (de)protonated ion may be less stable. However, when quantifying an analyte based on its adduct, a PCIS forming the same adduct would be preferable.

Considering these characteristics, fluorinated anandamide (2F-AEA) was selected as a PCIS candidate for DHEA, SEA, LEA, OEA, PEA and AEA. It was anticipated that NADA and 2-AG would probably require an alternative PCIS, but for this example we will focus only on a single PCIS, of which the following sections report the results.

### PCIS Concentration Optimization

After selecting the PCIS candidate, its signal intensity requires optimization. This intensity depends on both the flow rate of the postcolumn infusion and its concentration. Increasing the flow rate results in not only a higher PCIS intensity but also a dilution of the LC-flow and a loss of sensitivity for targets. Therefore, once a precise flow is achieved, it is recommended to optimize the concentration.

**Figure 2:**
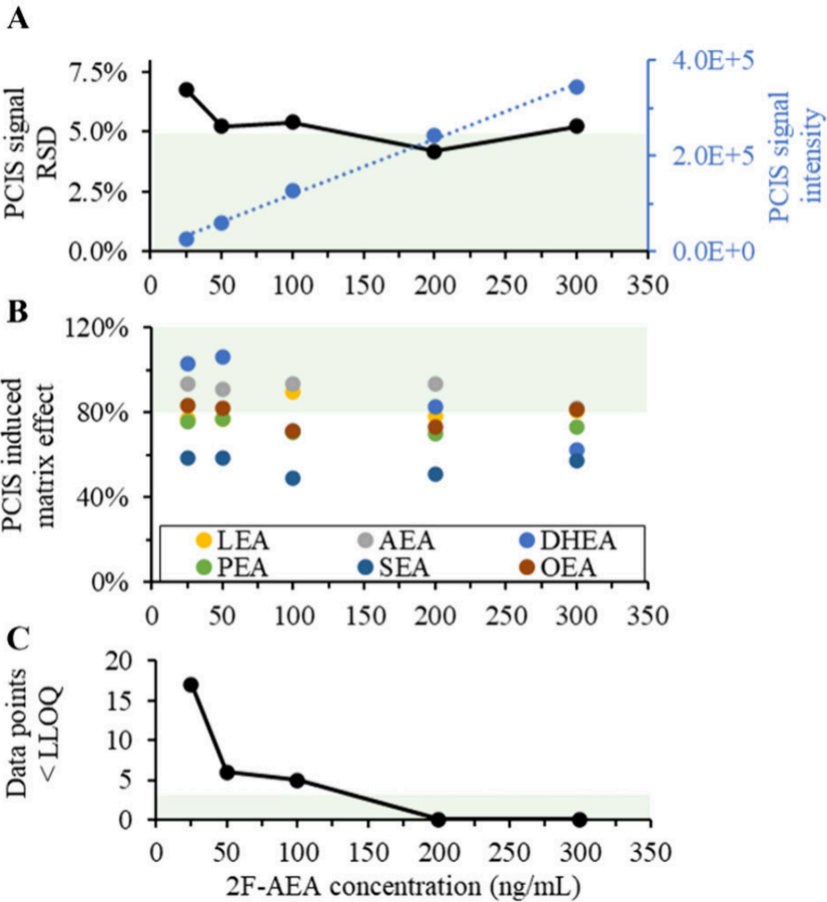
PCIS concentration optimization. The effect of the PCIS (2F-AEA) concentration on (A) its signal stability (RSD, in black) and mean signal intensity (blue, with linear regression line); (B) the mean matrix effect it causes on endocannabinoids in plasma (*n* = 3); (C) the number of data points dropping below the LLOQ. All plots share the same *x* axis, and values outside the green areas are suboptimal.

Generally, it is advisible to set the intensity level of the PCIS as low as possible (to minimize costs and matrix effect induced by the PCIS), but without compromising the stability of the signal and without the signal going below the LLOQ during a chromatographic run. The intensity level can be optimized by varying the PCIS concentration and measuring the RSD and the intensity of the PCIS signal when combined with an isocratic LC-flow, as shown in Figure 2A. The use of an isocratic flow prevents peaks or dips in the signal associated with the injected sample affecting the calculated RSD. As expected, the PCIS signal intensity linearly increased with the concentration. The RSD stabilized around 5% with concentrations at or above 50 ng/mL, but it is important to realize that the signal RSD is more related to its intensity than the concentration. It also means that if the PCIS intensity when injecting a biological sample is lower than during the isocratic infusion experiment (which could occur due to suppression), the PCIS concentration must be increased to reachieve the intensity level offering the desired stability.

The matrix effect caused by the PCIS and the number of data points below the LLOQ in the elution area of the analytes-of-interest also need evaluation to optimize the concentration. This requires the injection of biological samples while infusing the different PCIS concentrations. For the example method, as Figure 2B shows, the matrix effect caused by the PCIS showed a slight decreasing trend with increasing concentration, with a mean of 82% at 25 ng/mL to 73% at 300 ng/mL. It should be noted, that because no correction could be applied, there was high variation between the triplicate measurements. Still, we considered the matrix effect caused by the PCIS to be acceptable across all concentration levels. However, the number of data points below the LLOQ in the endocannabinoid elution area was >5 up to 100 ng/mL (Figure 2C). As each of these data points potentially disturbs the correction, a concentration of 150 ng/mL was selected as a compromise between costs, induced matrix effect and reliable signal measurement.

When doing method development with more than one PCIS, an equal-intensity (as opposed to equal-concentration) mix can be used at various levels to evaluate the matrix effect caused by the PCIS mix and to evaluate the number of data points below the LLOQ of the most suppressed signal.

### Evaluating the Ability of PCIS to Correct for Differences in Matrix Effect

**Figure 3:**
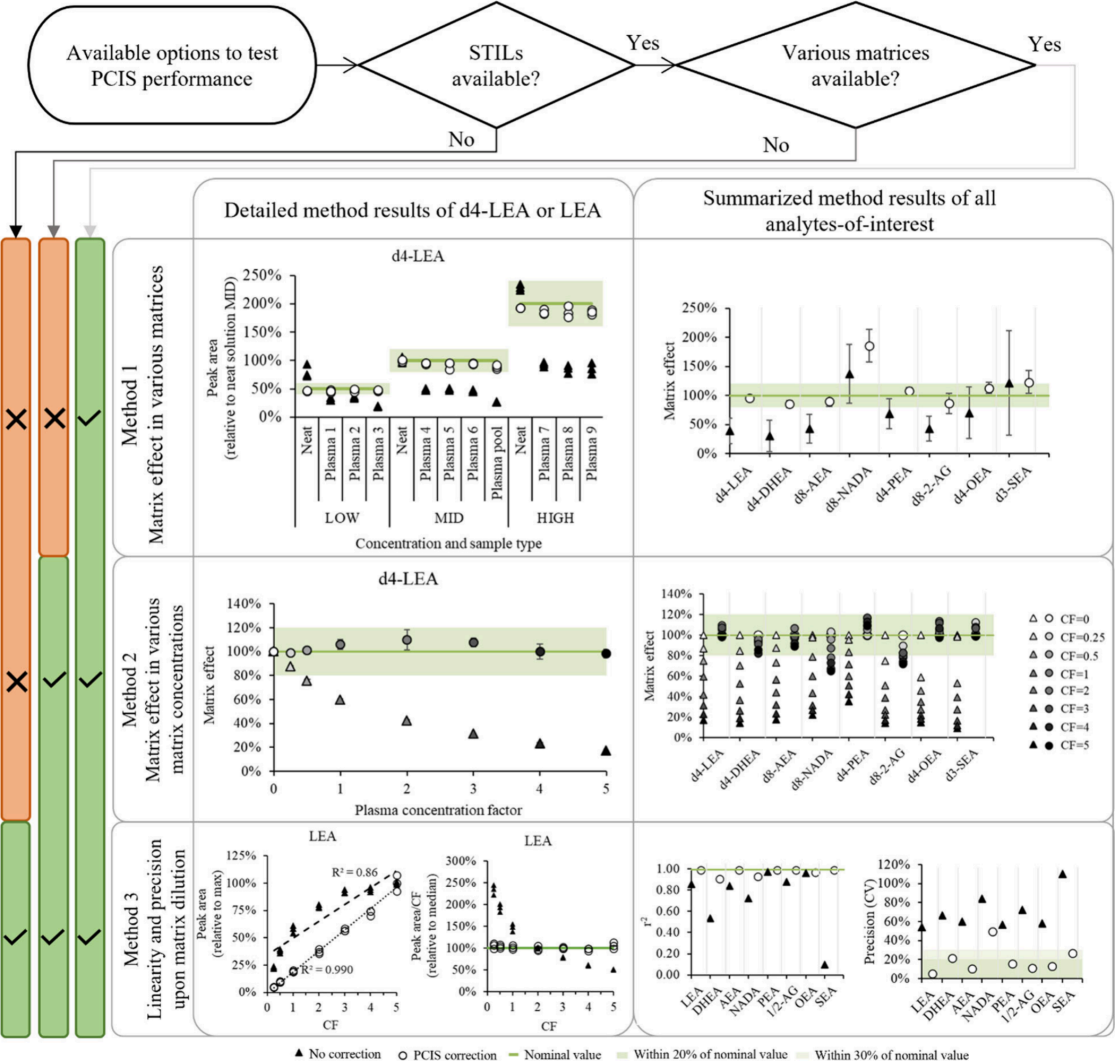
Flowchart of methods to evaluate the postcolumn infused standard (PCIS) performance and their results upon application to an LC-MS/MS method of endocannabinoids with 2F-AEA as a PCIS. STILs, stable isotope labeled internal standards; CF, concentration factor.

With the PCIS concentration optimized, the next step is to test its ability to correct. The best way for this is to induce a matrix effect by varying the matrix or its concentration and assess the ability of the PCIS to correct for this. Various methods exist to do this, and we will here present three alternatives using the endocannabinoid analysis as an example. The method of choice for future method development with PCIS will depend on the availability of SIL standards and diverse sources of matrix, as illustrated in the flowchart of Figure 3.

If diverse matrices and SILs are available, then the PCIS performance can be tested by calculating the matrix effect and precision across matrices, both with and without correction by the PCIS. The Method 1 pane in Figure 3 shows the results of such an analysis for the endocannabinoids. Here, 9 diverse plasma samples and the lab plasma pool were spiked (after sample preparation) with a “LOW” (50%), “MID” (100%) or “HIGH” (200%) concentration of deuterated endocannabinoids. The left plot of the pane shows that without correction, d4-LEA suffers from relative matrix effect (d4-LEA is more suppressed in plasma 3 and the plasma pool than in other samples) and nonlinearity (the relative peak area increase between the levels is not constant). Both issues are solved by PCIS correction as all corrected peak areas fall within the 20% region of the nominal value. The plot on the right gives the mean matrix effect and coefficient of variation (CV, also referred to as precision and visualized by the error bars) for each labeled endocannabinoid across all samples and levels. While this plot clearly shows that 2F-AEA is an unsuitable PCIS for d8-NADA, it also shows that for the other analytes, the matrix effect was improved from 30% - 121% to 85% - 123%, and the precision from 21% - 90% to 5% - 20%.

If the availability of different sources of matrix is limited, the concentration factor of a single matrix could be varied to test the ability of the PCIS to correct for the matrix effect induced by the increased matrix (Method 2 in Figure 3). When applied to the endocannabinoid example, plasma with a concentration factor (CF) between 5 and 0.25 (= four times diluted plasma) was spiked with SIL standards. As the left plot in the Method 2 pane shows for d4-LEA, the plasma CF clearly affected the uncorrected peak area. The matrix effect logarithmically decreased with increasing the CF, in line with previous studies.^27^ Looking at the summary plot at the right, 2F-AEA had the most difficulty to correct for NADA and 2-AG, agreeing with the initial exploration. For the other compounds the PCIS correction improved the matrix effect from 9% - 98% to 86% - 118%.

In absence of various sources of matrix and SIL standards, PCIS performance can be assessed by its ability to improve the linearity and the precision of the endogenous analytes across a dilution series (Method 3 of Figure 3). In this case, plasma was spiked with the compounds-of-interest, but interestingly, if the sensitivity to the endogenous analyte concentration allows, this is even possible without standards. When plotting the noncorrected peak area of LEA against the plasma CF, (left in the Method 3 pane) it gives a flattening curve with a coefficient of determination (r2) of only 0.86. The matrix effect causing this was effectively corrected using 2F-AEA as PCIS, as demonstrated by the increased r2 to 0.99. In the absence of matrix effect, dividing the peak area by the CF would give the same value across all CFs. This ratio is plotted for LEA, and it can again be observed that the matrix effect has effectively been corrected with 2F-AEA. The CV of these values indicates the precision of the dilution series and these, as well as the r2 values are plotted for each endocannabinoid at the right Method 3 pane. Excluding d8-NADA, which was not well corrected by the PCIS used, the range of r2 values improved from 0.10–0.97 to 0.9–0.99 and the precision from 54% - 110% to 5% - 27%.

Since these values might still seem suboptimal, a few points need to be taken into consideration. First of all, it should be noted that the acceptance criteria for endogenous compounds in a concentration series might be chosen less stringent than for usual calibration curves, as also proposed in the draft ICH guideline M10 on bioanalytical method validation^28^ advising a limit of 30% for the precision in a dilution series. The guideline describes dilution in blank matrix, but when measuring endogenous compounds, achieving these standards is even more difficult because of the different matrix for each sample in the calibration curve.

Method 3 has some more disadvantages. The concentration series is limited in the minimum concentration factor (because of sensitivity to the endogenous analyte) and its maximum (because of solubility issues during reconstitution into a smaller volume). In the presented example, the minimum concentration factor (0.25) still gave a considerable peak for PEA, but was around the detection limit for DHEA, causing high variability (22%) and a suboptimal r2 (0.90). Finally, Method 3 does not allow to test the performance of the PCIS in correcting for absolute matrix effect (which is a requirement for absolute quantification) due to lack of a neat solution with the same concentration. Due to these limitations, this method is probably more useful to compare the performance of different PCISs rather than assessing the performance of a single one.

### From Relative to Absolute Quantification

The value of metabolomics studies could be greatly enhanced if absolute concentrations would be reported. In the absence of an analyte-free matrix, to quantify analytes that are also endogenous compounds, calibration curves can be made using the standard addition method (SAM), background subtraction, surrogate analyte or surrogate matrix approach.^28^ The SAM requires multiple analyses of the same sample spiked at different levels making it time-consuming and impractical. The background subtraction method uses a pooled sample to build the calibration curve, meaning that part of the samples will have a concentration below the lowest calibration point requiring extrapolation of the curve. The surrogate analyte approach needs a surrogate analyte per endogenous analyte (preferably a SIL analogue) to build the calibration curve in matrix, resulting in extreme costs when measuring many analytes. Finally, the surrogate matrix approach, using an alternative matrix (e.g., a neat solution) for the calibration curves, also needs an ISTD per analyte, unless there is (virtually) no matrix effect. If PCIS could correct for this matrix effect, it becomes possible to quantify endogenous analytes based on a calibration curve in neat solution instead of matrix.

Before testing this, it is important to consider the limitations of PCIS. If working well, it can correct for matrix effect during ionization, but LC-MS/MS analyses have additional sources of variation. Correcting variability in sample preparation recovery or matrix effects other than during ionization (such as differences in target solubility or adsorption to vial surfaces between matrix and neat solution) still requires an ISTD spiked at the start of the sample preparation. To correct for sample injection volume, an external standard (ESTD) could be spiked in the last step of the sample preparation. When using PCIS correction, these spiked ISTDs or ESTDs should also be corrected with a PCIS. Instead of a spiked ISTD per analyte, a single ISTD is expected to correct for the sample preparation recovery of a whole range of analytes. With the aim of the present study being to show the potential of PCIS, sources of variability other than during ionization are not within the scope of this study and all samples were spiked after the sample preparation.

**Figure 4:**
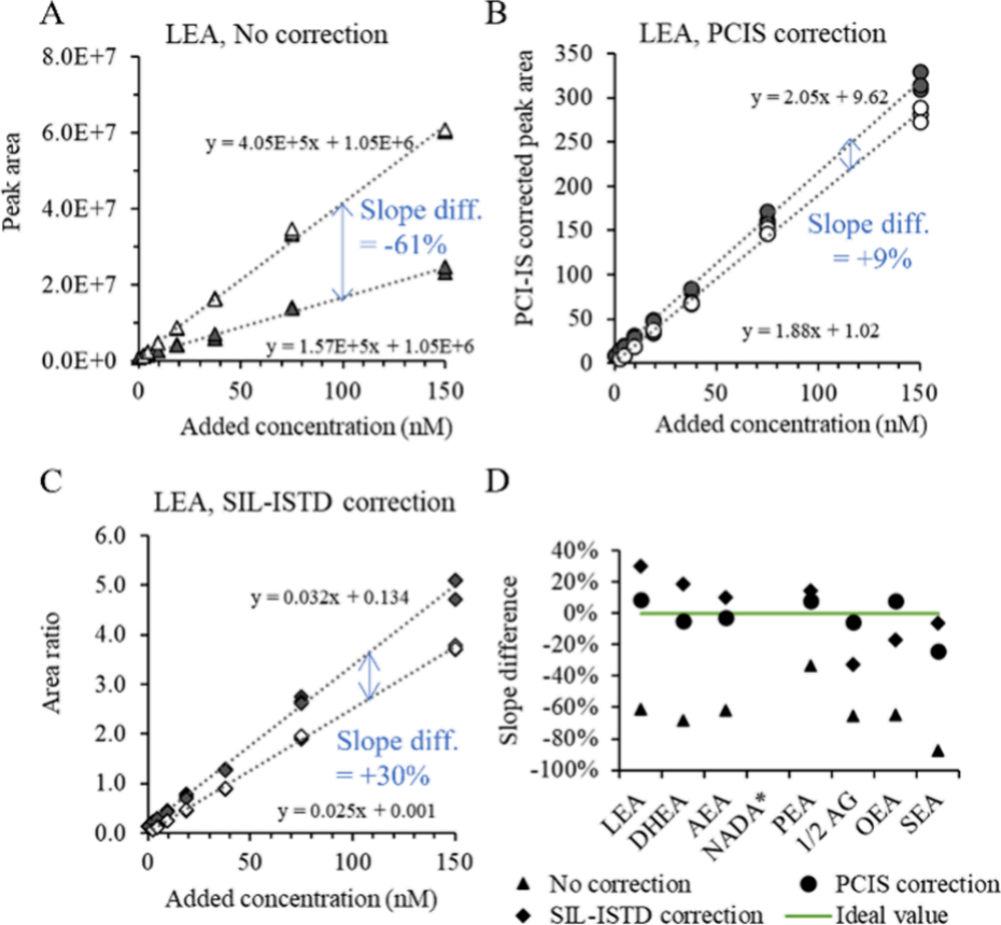
Evaluation of paralellism between calibration lines in plasma and neat solution. Linear regression of the calibration standards of LEA in neat solution and spiked to plasma without any correction (A), with postcolumn infused internal standard (PCIS) correction (B), and with spiked stable isotope labeled internal standard (SIL-ISTD) correction (C). Summarizing plot of the resulting differences in slope for LEA and the other endocannabinoids under investigation (D). *NADA gave a nonlinear curve in both plasma and academic samples, making comparison of slopes unreliable; therefore, this is not reported.

To justify the use of neat standards as a surrogate calibration line for matrix samples, parallelism should be evaluated,^28^ which is done by slope comparison in Figure 4. Figure 4A shows calibration curves for LEA in plasma and neat solution without any correction, and Figure 4B after correction with PCIS. For comparison, a traditional peak area correction with spiked SIL-ISTD LEA-d4 was performed (Figure 4C). The same was done for the other endocannabinoids and the resulting slope differences (% difference of the slope in plasma as compared to that in neat solution) are plotted in Figure 4D. This last figure reconfirms that the plasma matrix severely suppresses the signal of the endocannabinoids, as reflected by the slopes being 33% (PEA) up to 87% (SEA) lower in plasma as compared to neat solution. Quantifying these endocannabinoids without any correction based on the calibration curve in neat solution would result in a serious underestimation of the concentration.

Surprisingly, when comparing the slope differences between the PCIS and the SIL-ISTD in Figure 4D, the PCIS correction out- performed the correction using the spiked SIL-ISTD, which is considered the golden standard. Considering the high variability in suppression in this chromatographic area, the suboptimal performance of the SIL-ISTD correction may be due to the minor retention time difference between the analyte and its slightly more polar deuterated analogue.^29^ Indeed, when overlaying peaks of LEA and d4-LEA (see SI Figure S2) it becomes clear that LEA-d4 elutes slightly earlier and thereby suffers more from ion suppression, resulting in an overcorrection of the LEA peak (and a steeper slope of the calibration curve). These results indicate that in areas of serious and variable matrix effect, a perfectly coeluting structural analogue (like the PCIS) might correct even better for matrix effect than a spiked SIL-ISTD with a slight difference in retention time.

Taking into account the difficulty to correct the slopes for matrix effect even with a spiked SIL-ISTD, we consider a difference in slope up to ±15% preferable, but up to ±20% acceptable; the latter being a cutoff applied before by Godoy et al. comparing slopes of calibration curves of metabolites in serum and urine versus a synthetic surrogate matrix.^30^ The difference in slope of SEA (−24%) was outside of the acceptable limit. In the earlier tests, SEA, being the last eluting endocannabinoid and almost completely suppressed by the matrix, was also at the limits of the acceptance criteria. The slope differences of LEA, AEA, DHEA, PEA, OEA and even 2-AG easily fall within the preferable range (with a maximum of +9%). Therefore, these compounds could all be quantified using 2F-AEA as a PCIS to correct for matrix effect and calibration standards in neat solution.

## Conclusions

This study aimed to increase the appreciation and use of postcolumn infusion of standards (PCIS) as a tool to correct for matrix effect in LC-MS analysis, with the eventual goal to generate absolute quantitative data for metabolomics studies. To this end, we provided guidance in PCIS method development and performance testing, and we demonstrated its use with a LC-MS/MS method analyzing endocannabinoids in plasma.

With a simple infusion experiment it was estimated that at least six (LEA, AEA, DHEA, PEA, OEA and SEA) out of 8 (also including 2-AG and NADA) targeted endocannabinoids could be corrected with the same PCIS. Six characteristics of a promising PCIS candidate were defined and used to select 2F-anandamide (2F-AEA) to test as a PCIS for the endocannabinoids in this study. This choice was successful, as became clear after optimizing its concentration and applying three approaches to assess its performance, all leading to similar conclusions: the PCIS 2F-AEA was not successful in the matrix effect correction of NADA, but for the other analytes it could improve the matrix effect values for various matrices, the precision over matrices and over matrix concentrations, and the dilutional linearity and precision to within acceptable limits.

Keeping in mind that this study only considered variability due to matrix effect during ionization and a correction for variability in sample preparation and injection volume would still be advisible, we showed that using 2F-AEA as PCIS, LEA, AEA, DHEA, PEA, OEA and 2-AG could be quantified with good accuracy (slope difference with plasma < ±9%) using a calibration series in neat solution. In this case of severe and variable ion suppression PCIS correction performed even better than the “gold standard” correction with spiked SIL-ISTDs. With the endocannabinoid-related compounds in this study exemplifying a worst-case scenario in terms of matrix effect, the use of PCIS can be extended to other compound classes and other fields analyzing large numbers of analytes, such as exposome research.

Based on the wide applicability of the technique and the results it showed here, we conclude that PCIS is indeed a powerful tool for the necessary step toward absolute quantification in metabolomics.

## Supporting Information Available

Overview of compounds used (Table S1), LC-MS/MS conditions (Table S2), schematic drawing of instrument setup with PCIS (Figure S1), and PCIS correction outperforming SIL-ISTD correction due to retention time difference (Figure S2) (PDF)
