# Supplementary material for: Strategies for Using Postcolumn Infusion of Standards to Correct for Matrix Effect in LC-MS-Based Quantitative Metabolomics
Source: J Am Soc Mass Spectrom. 2024 Nov 15;35(12):3286–95. doi: 10.1021/jasms.4c00408 (PMC11622366; doi:10.1021/jasms.4c00408)

Supporting Information

"Strategies for Using Postcolumn Infusion of Standards to Correct for Matrix Effect in LC-MS-Based Quantitative Metabolomics"

Anne-Charlotte Dubbelman[a,b]*, Bo van Wieringen[a], Lesley Roman Arias[a], Michael van Vliet[a], Roel Vermeulen[b], Amy C. Harms[a], Thomas Hankemeier[a]*

[a]Metabolomics and Analytics Centre, Leiden Academic Centre for Drug Research (LACDR), Leiden University, Einsteinweg 55, 2333 CC Leiden, The Netherlands

[b]Institute for Risk Assessment Sciences, Department of Veterinary Science, Utrecht University, Yalelaan 1, 3584 CM, Utrecht, The Netherlands

*Corresponding authors

E-mail address Anne-Charlotte Dubbelman: a.c.dubbelman@uu.nl

E-mail address Thomas Hankemeier: hankemeier@lacdr.leidenuniv.nl

Table of Contents

Supplementary Table S1. Overview of structures and classifications of compounds used in this study. 3

Supplementary Table S2: LC-MS/MS conditions 4

Supplementary Figure S1: Schematic drawing of the LC-MS/MS instrument setup with PCIS 5

Supplementary Figure S2: PCIS correction outperforming SIL-ISTD correction due to retention time-shift. 6

Supplementary Table S1. Overview of structures and classifications of compounds used in this study.

Analyte (labeled)	Abbreviation (labeled)	Molecular structure (labeled)	Lipid maps ID [1] (unlabeled)	Sub class[1]
**Linoleoyl Ethanolamide-d4**	d4-LEA	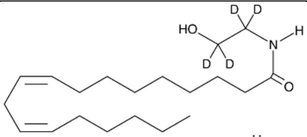	LMFA08040004	N-acyl ethanolamines (endocannabinoids) [FA0804]
**Docosahexaenoyl Ethanolamide-d4**	d4-DHEA	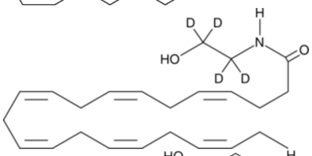	LMFA08040009	N-acyl ethanolamines (endocannabinoids) [FA0804]
**Arachidonoyl Ethanolamide-d8**	d8-AEA	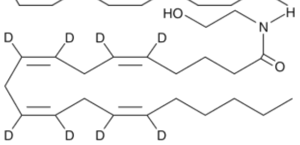	LMFA08040001	N-acyl ethanolamines (endocannabinoids) [FA0804]
**N-Arachidonoyl Dopamine-d8**	d8-NADA	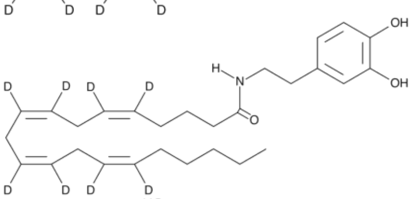	LMFA08020084	N-acyl amines [FA0802]
**Palmitoyl Ethanolamide-d4**	d4-PEA	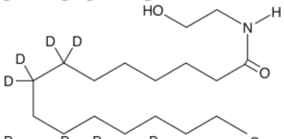	LMFA08040013	N-acyl ethanolamines (endocannabinoids) [FA0804]
**2-Arachidonoyl Glycerol-d8**	d8-2AG	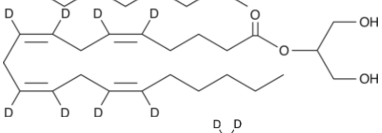	LMGL01010023	Monoacylglycerols [GL0101]
**Oleoyl Ethanolamide-d4**	d4-OEA	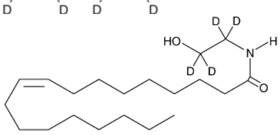	LMFA08040015	N-acyl ethanolamines (endocannabinoids) [FA0804]
**Stearoyl ethanolamide-d3**	d3-SEA	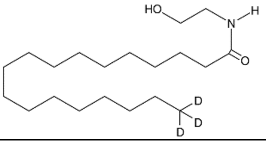	LMFA08040051	N-acyl ethanolamines (endocannabinoids) [FA0804]
**PCI-IS**	**Abbreviation**	**Molecular structure**	**Lipid maps ID (unlabeled)**	**Sub class (Lipid maps)**
**Arachidonoyl-2'-Fluoroethylamide**	2F-AEA	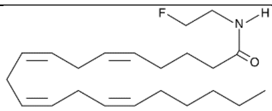	LMFA08020055	N-acyl amines [FA0802]

Reference:

1. Fahy, E. *et al.* Update of the LIPID MAPS comprehensive classification system for lipids. *J. Lipid Res.* **50**, 9–14 (2009).

Supplementary Table S2: LC-MS/MS conditions

**Autosampler temperature**	7 ºC
**Oven temperature**	40 ºC
**LC Flow rate**	0.7 mL/min
**Divert valve switched to waste**	0 – 0.8 min and 15 – 16 min
**Divert valve switched to MS**	0.8 – 15 min

Gradient

**Time**	**Module**	**Events**	**Parameter**
0.75	Pumps	Pump B Conc.	20%
0.95	Pumps	Pump B Conc.	26%
6	Pumps	Pump B Conc.	34%
7	Pumps	Pump C Conc.	1%
8	Pumps	Pump B Conc.	40%
10	Pumps	Pump B Conc.	54%
11	Pumps	Pump C Conc.	1%
12	Pumps	Pump B Conc.	56%
12	Pumps	Pump C Conc.	3%
13	Pumps	Pump B Conc.	78%
13	Pumps	Pump C Conc.	6%
14	Pumps	Pump B Conc.	85%
14	Pumps	Pump C Conc.	15%
14.5	Pumps	Pump B Conc.	85%
14.5	Pumps	Pump C Conc.	15%
14.8	Pumps	Pump B Conc.	20%
14.8	Pumps	Pump C Conc.	1%
16	System controller	Stop	

General MS parameters

**Experiment type**	MRM
**Curtain gas**	40
**Gas 1**	50
**Gas 2**	50
**Ion Spray voltage**	4500
**Temperature**	600 ºC

Compound specific MS parameters of the measured endocannabinoids and PCI-IS

**Name**	**Q1**	**Q3**	**Rt center (min)**	**Rt width (s)**	**CE**	**CXP**	**DP**	**EP**
d4-LEA	328.300	66.200	12.9	30	36	11	74	10
d4_DHEA	376.300	66.200	12.9	30	25	11	70	10
LEA	324.000	62.000	12.9	30	36	11	74	10
AEA	348.000	62.000	13	30	38	11	72	10
d8-AEA	356.300	62.200	12.9	30	42	11	70	10
DHEA	372.000	62.000	12.9	30	25	11	70	10
d8-2-AG	387.300	294.200	13.2	30	20	11	56	10
1/2-AG	379.210	287.000	13.2	30	19	11	56	10
d4-PEA	304.300	62.200	13.2	30	36	11	78	10
PEA	300.000	62.000	13.2	30	36	11	78	10
d3-SEA	331.300	62.200	13.7	30	25	11	72	10
SEA	328.000	62.000	13.7	30	31	11	72	10
NADA	440.000	137.000	13.3	30	34	11	75	10
d8-NADA	448.400	137.000	13.3	30	34	11	75	10
OEA	326.000	62.000	13.4	30	30	11	72	10
d4-OEA	330.300	66.200	13.4	30	30	11	72	10
2F-AEA	350.300	269.200	7.5	900	20	11	50	10

Supplementary Figure S1: Schematic drawing of the LC-MS/MS instrument setup with PCIS

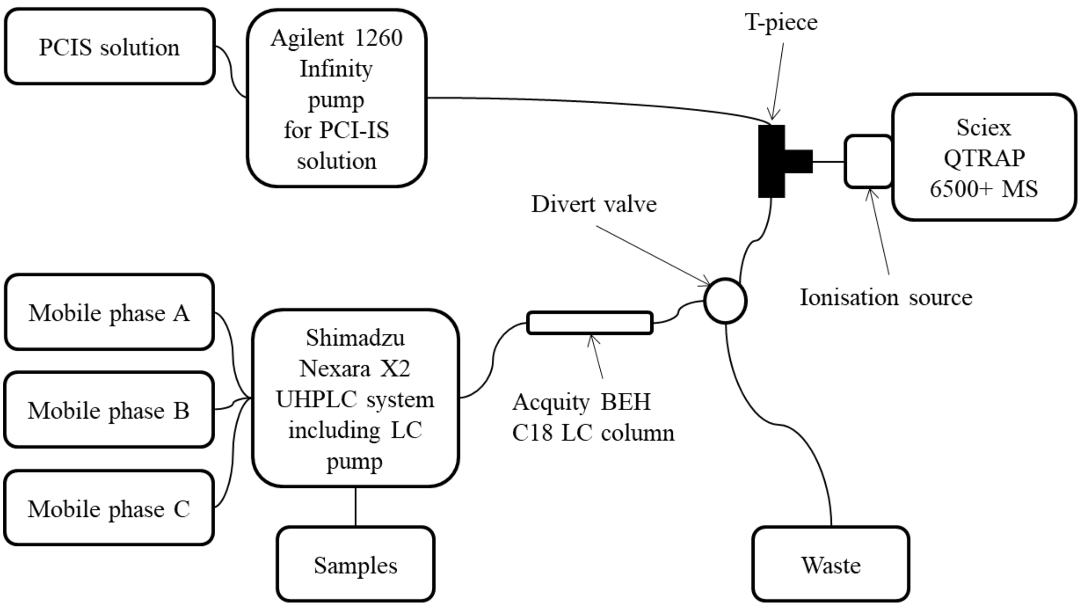

Supplementary Figure S2: PCIS correction outperforming SIL-ISTD correction due to retention time-shift.

Examples of retention time difference between a deuterated standard and its non-labeled analogue in a region of variable ion suppression, shown by overlaid MRM-transitions of the PCIS (grey), a deuterated standard (blue) and its non-labeled analogue (orange) in a plasma calibration standard. A: d4-LEA elutes earlier than LEA and is more affected by ion suppression (around 12.9 min). The d4-LEA peak is therefore smaller than it would be if the ion suppression was the same as for LEA, resulting in an overcorrection when using d4-LEA to correct for matrix effect by peak area correction. B: d8-2AG elutes earlier than 2-AG and suffers less from ion suppression (around 13.3 min). The d8-2-AG peak is therefore larger than it would be if the ion suppression was the same as for 2-AG, resulting in an undercorrection when using d8-2-AG to correct for matrix effect by peak area correction.

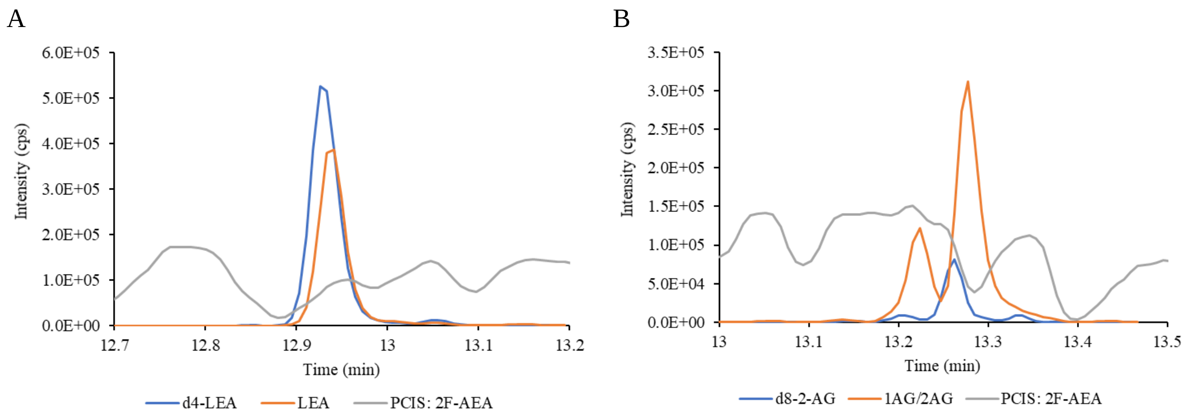

Supplement: Supplementary file 1 — js4c00408_si_001.pdf [file js4c00408_si_001.pdf]
